# Preparation and physical properties of a Cr_3_Al film with a DO_3_ structure

**DOI:** 10.1107/S2052252519004469

**Published:** 2019-05-07

**Authors:** W. Q. Zhao, X. F. Dai, X. M. Zhang, Z. J. Mo, X. T. Wang, G. F. Chen, Z. X. Cheng, G. D. Liu

**Affiliations:** a School of Materials Science and Engineering, Hebei University of Technology, Tianjin 300130, People’s Republic of China; b School of Materials Science and Engineering, Tianjin University of Technology, Tianjin 300384, People’s Republic of China; c Institute for Superconducting and Electronic Materials (ISEM), University of Wollongong, Wollongong 2500, Australia

**Keywords:** electrical transport, structural properties, magnetron sputtering, crystallization, crystal growth, density functional theory, materials science

## Abstract

A Cr_3_Al compound with a DO_3_ structure has previously been predicted to be nearly half metal and a promising spintronics material; however, its synthesis has not been reported. Here, a Cr_3_Al compound with a DO_3_ structure is successfully prepared in thin-film form by the magnetron sputtering method. It was found that the substrate temperature is crucial to the atomic ordering, thin-film density and lattice constant. The lattice constant varies with different substrate temperatures and is smaller than the theoretical equilibrium lattice constant. Theoretical investigations on the electronic structures and magnetic properties indicate that the Cr_3_Al compound with a DO_3_ structure is a rare material with zero-gap half-metallic characteristics under an experimental lattice constant of 5.83 Å. The experimental result is in agreement with the theoretical results in magnetization, and the Cr_3_Al compound synthesized in this work exhibits semi-metallic-like electrical transport characteristics and positive magnetoresistance of greater than 2% in the temperature range 2–250 K.

## Abstract 2

A Heusler Cr_3_Al thin film with a perfect DO_3_ structure is successfully prepared for the first time and its physical properties are investigated.

## 1. Introduction   

Materials that can supply highly spin polarized electrons or current are strongly desired in spintronics devices because the performance of such devices greatly depends on the spin polarization of the current or some key materials. The half-metallic materials (HMs) are generally characterized by a metallic band structure for one spin channel and a semiconductive/insulative band structure for the other spin channel. Such distinctive electronic band structures of HMs leads to a 100% spin polarization of electrons at the Fermi level (*E*_f_) and makes HMs one the most promising spintronics materials (Katsnel’son & Irkhin, 1994 ▸; Žutić *et al.*, 2004 ▸; Dieny *et al.*, 1991 ▸; Schmidt *et al.*, 2000 ▸; Fiederling *et al.*, 1999 ▸; DeGroot *et al.*, 1983 ▸). In recent years, a type of special HM [also called spin-gapless semiconductors (SGS)] has attracted more attention. For SGS, there is a zero gap at the Fermi level in the majority spin channel and the mobility of carriers is stronger than that in common semiconductors and not only the electrons but also the holes can be fully spin-polarized (Wang, 2008 ▸; Wang *et al.*, 2016 ▸, 2010 ▸; Zhang *et al.*, 2015 ▸; Xu *et al.*, 2013 ▸).

The concept of SGS was first proposed in the diluted magnetic Co-doped PbPdO_2_ by Wang *et al.* (2008 ▸). In 2013, Gao and Yao theoretically designed several high-spin-polarization materials in binary DO_3_-type structural *X*_3_ *Z* (*X* = Sc, Ti, V, Cr, Mn, Fe; *Z* = Al, Si, Ga, Ge) compounds. Their calculation results show that Cr_3_Al compounds have a high spin polarization at the Fermi level in a wide range of lattice constant and can be an SGS when the lattice constant is up to 6.22 Å (Gao & Yao, 2013 ▸). The Cr_3_Al compound with a DO_3_ structure is one of the few binary alloys with high polarization and SGS characteristics reported in previous theoretical studies. The experiments on the synthesis and physical properties of the Cr_3_Al film were carried out in 2012 by Boekelheide and coworkers. They used electron-beam evaporation to prepare Cr_3_Al thin films, but no samples with a perfect DO_3_ structure were successfully synthesized. Boekelheide pointed out that the *X* phase (in fact, the *X* phase is just a DO_3_ structure with an inhomogeneous distribution of Al atoms) is the most stable and was synthesized as a film by the electron beam evaporation method using a Cr_3_Al alloy. At the same time, the magnetic moment of the *X* phase was found to be far lower than that of the DO_3_ structure for the Cr_3_Al compound, which is consistent with theoretical predictions (Boekelheide *et al.*, 2012*a*,*b* ▸ ▸, 2010 ▸).

The unsuccessful experimental synthesis, the diverse band structure and physical properties of the Cr_3_Al compound with a DO_3_ structure predicted in previous work motivate us to carry out further experimental and theoretical investigations. In this paper, we attempted to prepare a Cr_3_Al film using the magnetron sputtering method. The Cr_3_Al compound with a DO_3_ structure has been successfully synthesized in thin-film form. We will show that a practical lattice constant of 5.83 Å is smaller than the theoretical equilibrium lattice constant. The electronic structure will also be calculated and discussed for the Cr_3_Al compound with a DO_3_ structure under a practical lattice constant of 5.83 Å. We will show that the Cr_3_Al compound with such a smaller lattice constant is a material with the rare zero-gap half-metallic characteristics. The experimental result is in agreement with the theoretical result in magnetization. The semi-metallic-like electrical transport characteristics and large positive magnetoresistance are observed and discussed for the Cr_3_Al compound with a DO_3_ structure in this paper.

## 2. Experimental and computational details   

Cr_3_Al films were prepared by an adjustable three-target ultra-high vacuum magnetron sputtering system with a base pressure below 3 × 10^−4^ Pa. The Cr and Al targets used to prepare Cr_3_Al compound films are made from pure metals with a purity higher than 99.9%. The substrates used in this work are 25 × 14 mm glass slides. Cr_3_Al compound films were deposited from two-target co-sputtering. The Ar pressure during sputtering was 1.0 mTorr and the deposition rate was about 0.5 nm s^−1^. The substrate temperature is selected at 50–400°C and the sputtering time is 30 min. In the rest of the paper, we will use ‘sample 100, sample 150…’ to denote the samples prepared at substrate temperatures of 100, 150°C *etc*. The structural properties of the sample were investigated by X-ray diffraction (XRD) using Cu *K*α radiation. The magnetic properties were detected by a vibrating sample magnetometer. The M–H curve of the sample with substrate was measured first and then the film was peeled off the substrate and the M–H curve of the substrate was subsequently measured. Finally, the M–H curve of the pure film was achieved by deducting the data of the substrate from the M–H curve of the sample with substrate. Surface morphology was observed using a scanning electron microscope (SEM). SEM was also used for energy-dispersive X-ray spectroscopy (EDS) to ensure the chemical composition of the film had not deviated from the Cr_3_Al stoichiometry. We used two methods to determine the density of the unit cell. One is in terms of the lattice constant achieved from the X-ray diffraction patterns. The other is according to the practical measurement of the mass and volume. The mass was measured by an electronic balance with an accuracy of 0.00001 g, which gave an error of less than 1% for the mass measurement of our film samples. The film thickness measurement of the sample was performed using a Dektak 6M-Stylus Profiler film thickness tester. The electronic transport measurements were performed using a physical property measurement system.

The electronic structure calculations were performed using the *WIEN2K* package based on the full-potential linearized augmented plane-wave method (Blaha *et al.*, 1990 ▸; Saini *et al.*, 2013 ▸; *Elk* FP-LAPW code, version 1.4.22 http://elk.sourceforge.net). The generalized gradient approximation in the Perdew–Bueke–Ernzerhof scheme was used to implement electronic-exchange related functions (Hafner *et al.*, 2002 ▸; Graf *et al.*, 2011 ▸; Hsu *et al.*, 2002 ▸; Perdew *et al.*, 1996 ▸). The muffin-tin radius used in the calculations is generated by the system. A converged ground state was obtained using 10000 *k* points in the first Brillouin zone with *K*_max_ *R*_MT_ = 8.0 (*K*_max_ represents the maximum size of the reciprocal-lattice vectors and *R*_MT_ is the muffin-tin radius). Wavefunctions and potentials inside the atomic sphere are expanded in spherical harmonics up to *l* = 10 and 4, respectively.

## 3. Results and discussion   

### 3.1. Morphology and structure characterization of the Cr_3_Al film   

**Figure 1:**
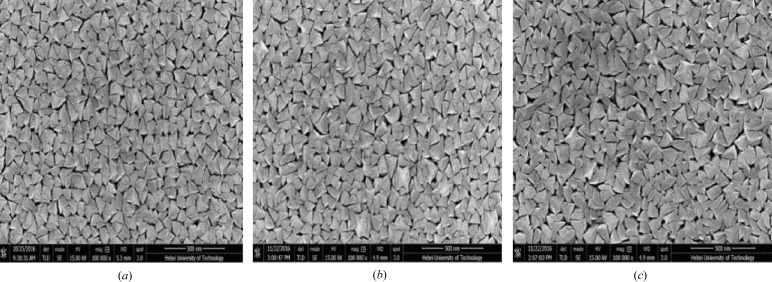
SEM images of (*a*) sample 100, (*b*) sample 150 and (*c*) sample 200.

Surface morphology of samples 100, 150 and 200 were observed by SEM and the images obtained of the sample surface are shown in Fig. 1 ▸. It is very clear that all the samples show the grain structure. From the perspective of overlooking the film surface, a single grain is in a triangular shape with sharp edges and a size of about 150 nm. The size of the grains are essentially uniform in the films. Thickness measurements show that the grain distribution and the film thickness are homogeneous in several square centimetres. The size of grains is similar and the space between grains has no obvious difference for samples 100, 150 and 200. The EDS shows the composition of the grains to be Cr_3_Al, with an uncertainty of ∼3% with respect to each element, *i.e.* very close to the intended stoichiometry for all the used sample in this paper. Detailed results on the film composition are shown in Table 1 ▸.

**Figure 2:**
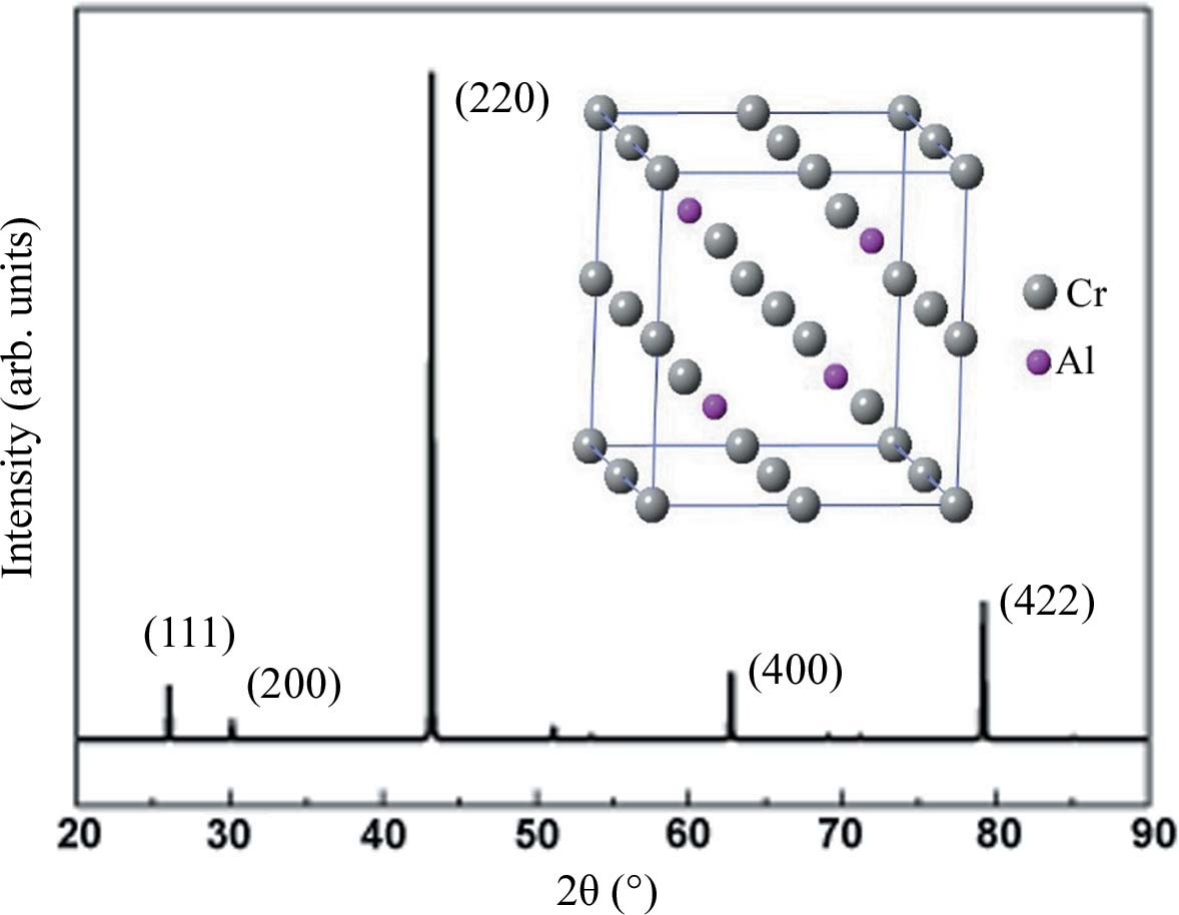
Theoretical powder XRD pattern for the Cr_3_Al compound with a DO_3_ structure; the inset is the structure model.

The DO_3_ structure has an 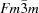 space group (No. 225) and can generally be seen as a set of four interpenetrating face-centered-cubic (f.c.c.) lattices, with A (0, 0, 0) B (1/4, 1/4, 1/4) C (1/2, 1/2, 1/2) and D (3/4, 3/4, 3/4) arranged along the space diagonal in the Wyckoff coordinates. For the Cr_3_Al compound with a DO_3_ structure, the Cr atoms occupy A, B and C sites and Al atoms occupy D sites as illustrated in the inset of Fig. 2 ▸. The order-independent principal peak reflections (220), (400) and (422) can be observed in the standard XRD powder diffraction patterns for a material with a perfect DO_3_ structure and the (111) and (200) peaks that correspond to the order-dependent superlattice reflections are much weaker in intensity than the principal peaks as shown in Fig. 2.

**Figure 3:**
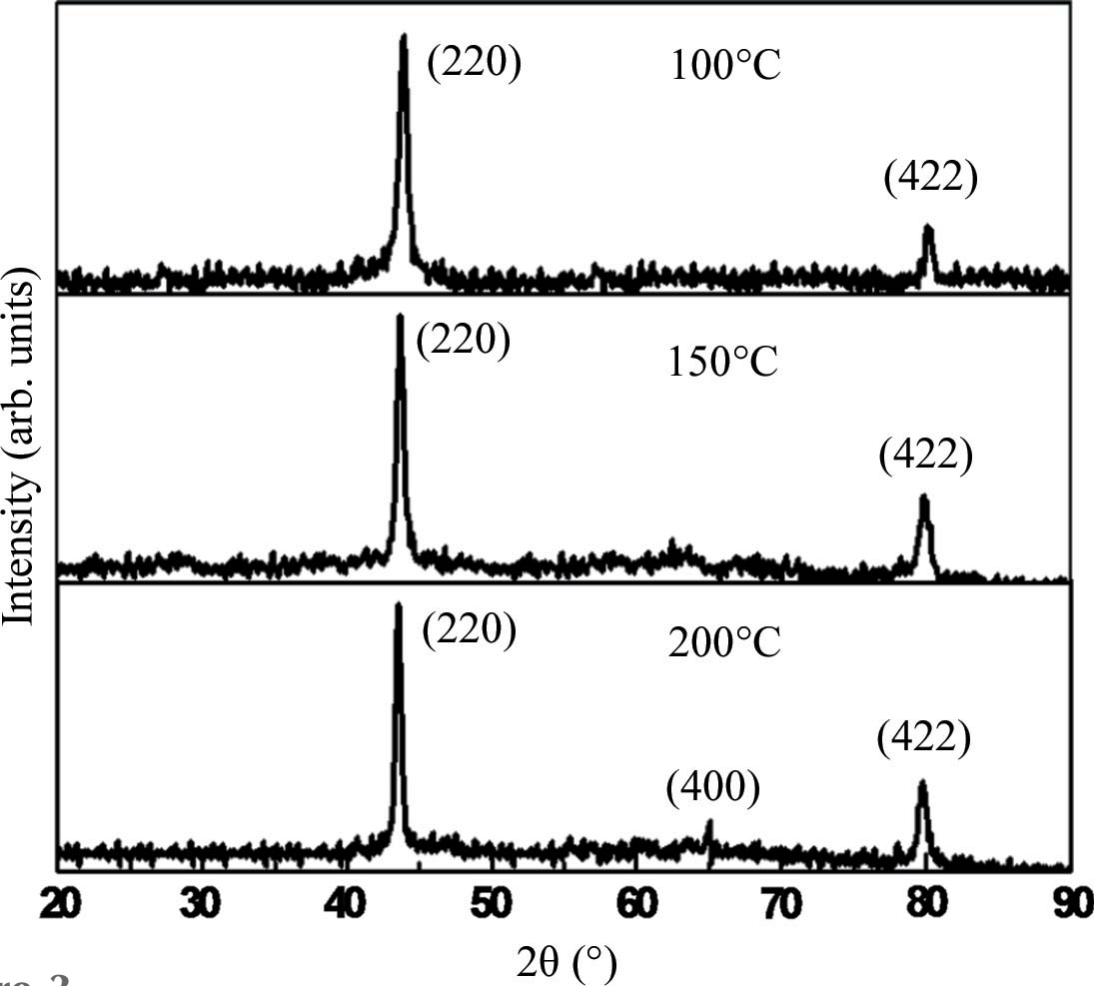
X-ray diffraction patterns of samples 100, 150 and 200.

Fig. 3 ▸ shows the XRD patterns of the Cr_3_Al film samples prepared at different substrate temperatures. The XRD patterns shown here are quite similar to the results reported by Boekelheide *et al.* (2012*a*) where the film samples were prepared by electron beam evaporation. The structure determined using XRD patterns is f.c.c. based and the order-independent principal peak reflections of (220) and (422) can be clearly observed for all samples prepared at different substrate temperatures. Also, no peaks of impure phases were observed in any of the XRD patterns. The order-independent principal peak of (400) is not observed due to its own very weak intensity and the effects of texture and peak widening originating from the thin-film form. The (111) and (200) peaks correspond to the order-dependent superlattice reflections and are several times weaker than (400) in intensity. As a result, they cannot be observed in XRD patterns. Even so, our XRD patterns can still show that all the samples crystallize in a pure phase with a DO_3_-structure framework, although the lack of (111) and (200) diffraction peaks lead to an uncertainty in the ordering degree of Cr–Al. Comparing the intensities of the (220) peak with the (422) peak, one can see that the crystal grains of sample 100 have a greater preference for the (220) orientation than those of sample 200. So, it is possible that sample 100 has no (400) diffraction peak in the XRD pattern, instead, sample 200 has this peak. As shown in previous work, the magnetization and magnetic structure are quite sensitive to the ordering degree of Cr–Al (Boekelheide *et al.*, 2012 ▸ *a*,*b* ▸, 2010 ▸). So, with the help of the magnetic measurements, we can determine the atomic ordering degree in the Cr_3_Al film, which will be investigated and discussed in detail in Section 3.2.

The lattice constants determined here are 5.83, 5.88 and 5.91 Å, and the film thicknesses are 8542.5, 9598.6 and 10068.6 Å for samples 100, 150 and 200, respectively. This indicates that the lattice constant increases and the Cr_3_Al compound density increases with increasing substrate temperature. Furthermore, when the substrate temperature is higher than 200°C, the lattice constant is stable at 5.91 Å, and the film thickness is also almost unchanged with changing substrate temperature. The data on the lattice constant and film thickness are also collected in Table 1 ▸. All the lattice constants are smaller than the theoretically predicted equilibrium lattice constant of 5.92 Å (Gao & Yao, 2013 ▸). In the work by Boekelheide *et al.* (2012*a*), the lattice constant is reported to be 5.90 Å for the Cr_3_Al compound film prepared by electron-beam evaporation (sample EBE), which is fairly consistent with the results determined in sample 200 or the samples prepared at substrate temperatures higher than 200°C. In fact, the magnetization is also very close for the reported sample EBE and our sample 200, which will be shown and discussed in the next section.

### 3.2. Magnetic properties and electronic structure   

**Figure 4:**
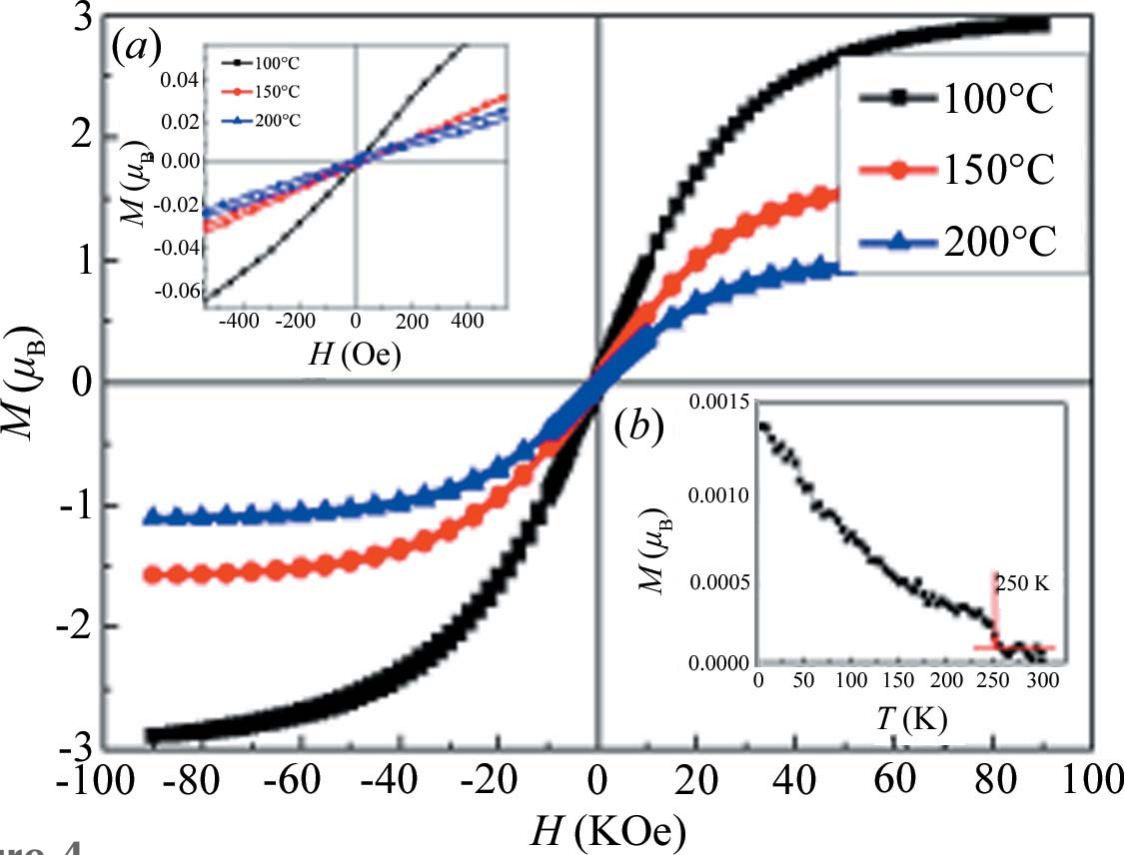
Magnetic field dependence of magnetization for samples 100, 150 and 200 measured at 2 K. (*a*) *M*–*H* curve tested under low field. (*b*) *M*–*T* curve of the sample.

Fig. 4 ▸ shows the magnetization curves measured at 2 K for samples 100, 150 and 200. The total magnetic moments of the unit cell (*M*_t_) achieved from the magnetization curves are shown in Table 1 ▸. *M*_t_ is 1.1 μ_B_ for sample 200, and was reported to be 1.06 ± 0.02 μ_B_ for sample EBE in Boekelheide *et al.* (2012*a*). It is clear that *M*_t_ is comparable for sample 200 and sample EBE. It is reported that sample EBE does not crystallize in a perfect DO_3_ structure but in an *X*-phase structure. The inhomogeneous distribution of Al atoms in Cr_3_Al causes the antiferromagnetic arrangement of the Cr atomic magnetic moment and a decrease in magnetization (Boekelheide *et al.*, 2012 ▸ *a*,*b* ▸, 2010 ▸). According to first-principles calculations, Cr_3_Al with a perfect DO_3_ structure should have an *M*_t_ of 2.92–3 μ_B_ when the lattice changes in the range 5.90–6.22 Å (Gao & Yao, 2013 ▸). To associate this with the lattice constant mentioned in Section 3.1 would imply that sample 200 has the same atomic occupation and ordering as sample EBE.

Furthermore, from Fig. 4 ▸ it can be seen that the saturation magnetization (namely, *M*_t_) increases with decreasing substrate temperature. When the substrate temperature reaches 100°C, the samples show an *M*_t_ of about 2.88 μ_B_. The *M*_t_ of 2.88 *μ_B_* is slightly smaller that the theoretical results reported by Gao & Yao (2013 ▸), where the Cr_3_Al compound is hypothetically in a perfect DO_3_ structure with a lattice constant of 5.90–6.22 Å. In fact, it should be noted that the lattice constant contracts with decreasing substrate temperature. For sample 100, the lattice constant is 5.83 Å, which is smaller than that used in the work by Gao & Yao (2013 ▸). In order to compare the experimental and theoretical results more clearly and accurately, the electronic structure and magnetic properties were calculated by first-principles calculations for the Cr_3_Al compound with a perfect DO_3_ structure and a lattice constant of 5.83 Å. The calculated atomic magnetic moments are 1.98 μ_B_, −1.29 μ_B_ and 0.03 μ_B_ for Cr (A, C), Cr (B) and Al atoms, and the *M*_t_ is 2.88 μ_B_ which is quite consistent with our experimental result of 2.88 μ_B_. This indicates that the Cr_3_Al compound with a perfect DO_3_ structure was successfully synthesized as a film at the substrate at 100°C by the magnetron sputtering method. In addition, Fig. 4(*a*) shows an enlarged part of the M–H curves at low field. One can see that the coercivity of Cr_3_Al is very small and Cr_3_Al has a fairly soft magnetic characteristic. Fig. 4(*b*) shows the temperature dependence of magnetization (M–T curve) in the field of 500 Oe. It is clear from the M–T curve that the Curie temperature of Cr_3_Al is 250 K.

**Figure 5:**
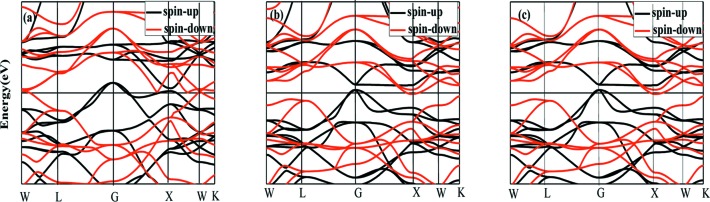
The calculated band structures for the Cr_3_Al compound with a DO_3_ structure at a lattice constant of (*a*) 5.83 Å, (*b*) 5.92 Å (equilibrium lattice constant) and (*c*) 6.22 Å.

The calculated band structures for the Cr_3_Al compound with a lattice constant of 5.83 Å are shown in Fig. 5 ▸. For comparison, we also provide the band structures at the equilibrium lattice parameter and at a lattice parameter of 6.22 Å. It is clear that these band structures are very similar. However, it can also be seen that at the lattice constant of 5.83 Å, the Cr_3_Al compound with a perfect DO_3_ structure is no longer a common half-metal or spin-gapless semiconductor for the Fermi level across the valence band top and the vanishing of the band gap in the spin-up channel. More importantly, such a band structure is characteristic of a zero-gap HM; hence, the Cr_3_Al compound synthesized in this work can be considered to be a zero-gap HM. The concept of a zero-gap HM was first proposed by Du *et al.* (2013 ▸). So far, the zero-gap half-metal as a special spintronics material has rarely been reported. However, many novel physical properties occurring in the zero-gap HM – for example, the crossover of magnetoresistance – have considerable theoretical research value and potential applications in the field of spintronics.

### 3.3. Electrical transport properties   

**Figure 6:**
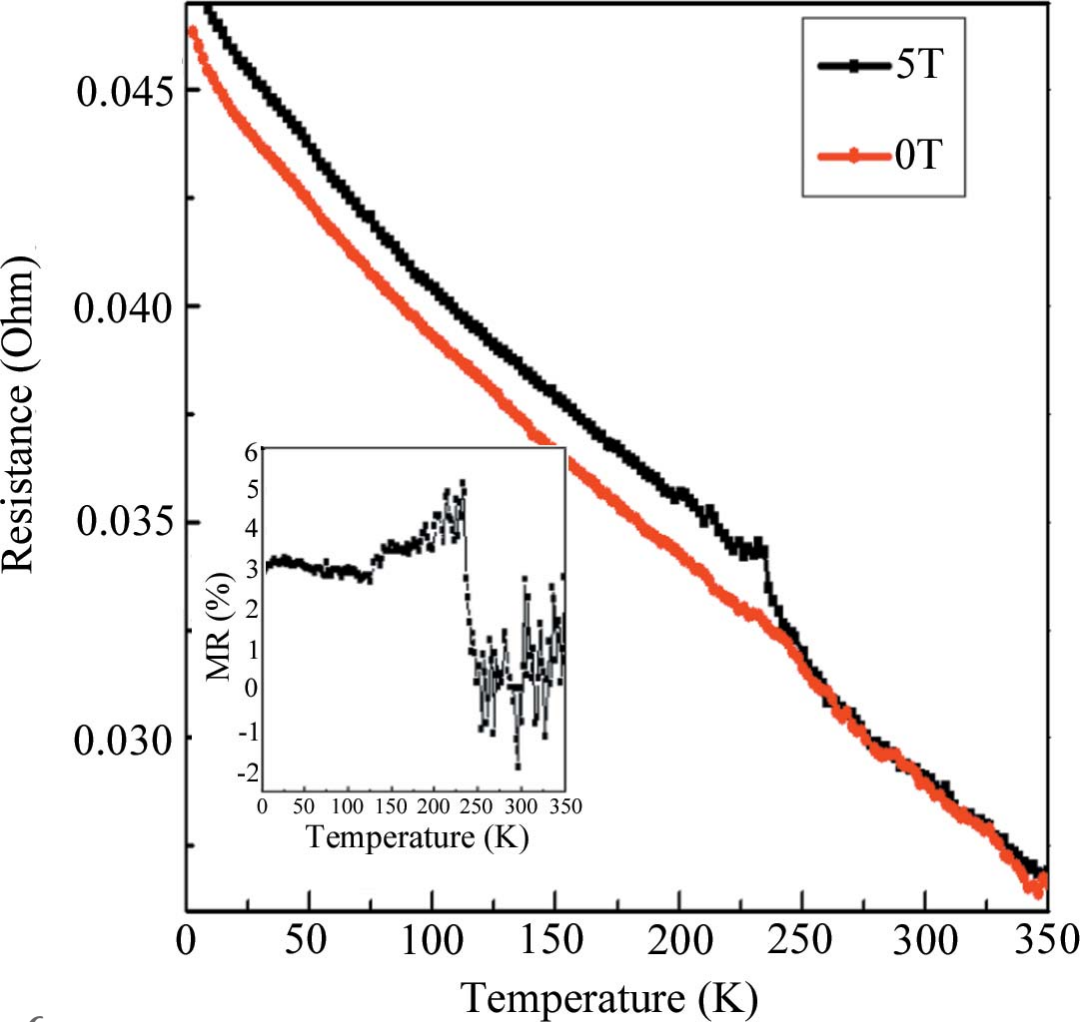
Dependence curves of resistance on temperature with and without a magnetic field for sample 100. The red line is the curve without an external magnetic field and the black line is the result at an external magnetic field of 5 T. The inset shows the magnetoresistance at different temperatures calculated from the dependence curves of resistance with and without a magnetic field.

**Figure 7:**
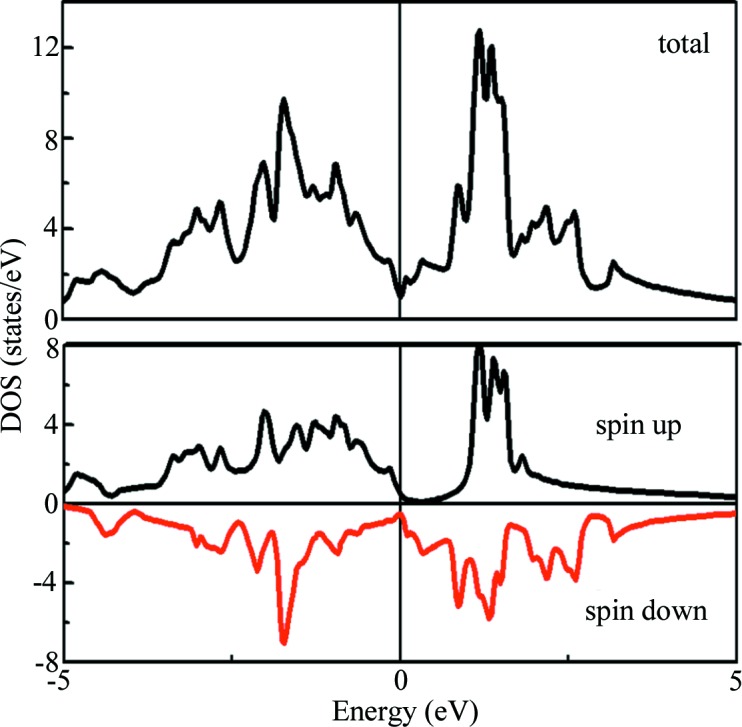
Calculated total DOS and spin-projected DOS patterns for the Cr_3_Al compound with a DO_3_ structure at a lattice constant of 5.83 Å.

Fig. 6 ▸ shows the dependence curves of resistance on temperature with and without the magnetic field for sample 100. It can be seen that the resistance of the Cr_3_Al compound decreases with increasing temperature and exhibits a semi-metallic-like behavior. The inflection points occurring near 250 K correspond to the Curie temperature achieved from the M–T curve. The total DOS and spin-resolved DOS patterns are shown in Fig. 7 ▸. It can be observed that the Fermi level always lies in a deep valley for spin-up, spin-down or the total DOS. Usually this deep valley is considered as a pseudo-gap and the semi-metallic-like electrical transport behavior can be attributed to the pseudogap-type electronic structure for Cr_3_Al compounds. Below the Curie temperature, the resistance with the magnetic field of 5 T is obviously higher than that without a magnetic field, which indicates a positive magnetoresistance behavior and the related data are plotted in Fig. 6 ▸(*b*). The positive magnetoresistance is higher than 2% and the maximum of about 5% occurs near the Curie temperature. Usually a positive MR can be widely observed in nonmagnetic systems. However, the special cases are the zero-gap half-metals and the spin-gapless semiconductors (Du *et al.*, 2013 ▸; Ouardi *et al.*, 2013 ▸). Du *et al.* (2013 ▸) and Ouardi *et al.* (2013 ▸) reported the positive MR of Fe_2_CoSi and Mn_2_CoAl Heusler compounds at low temperature and attributed this characteristic to the unique band structures, that is, spin-gapless semiconductive or zero-gap half-metallic band structures. As illustrated on the basis of band structures of Cr_3_Al shown in Fig. 5 ▸, the Cr_3_Al compound with the experimental lattice constant has a zero-gap half-metallic band structure. Therefore, the large positive magnetoresistance can be thought to be closely related to the zero-gap half-metallic characteristics of the band structure for the Cr_3_Al compound.

## 4. Conclusions   

A Cr_3_Al compound with a DO_3_ structure has been successfully synthesized in the film form by the magnetron sputtering method. An experimental lattice constant of 5.83 Å is achieved and is smaller than the theoretical equilibrium lattice constant. According to first-principles calculations, it was found that the Cr_3_Al compound with a DO_3_ structure is a material with rare zero-gap half-metallic characteristics in a much smaller lattice constant. The experimental result is in agreement with the theoretical result in magnetization. The semi-metallic-like electrical transport characteristics and large positive magnetoresistance are considered to originate from the special electronic structures of the Cr_3_Al compound with a smaller lattice constant.

## Figures and Tables

**Table 1:** The film thickness, density, composition (atomic ratio), experimental lattice constant (*a*) and magnetic moment of the unit cell (*M*_t_) for samples 100, 150 and 200

	Film thickness (Å)	Density (×10^3^ kg m^−3^)	Atomic ratio	*a* (Å)	*M*_t_ (μ_B_)
Sample 100	8494.2	6.08	3.00:1	5.83	2.88
Sample 150	9518.3	5.72	2.98:1	5.88	1.65
Sample 200	10088.6	5.29	2.99:1	5.91	1.10
